# Microbial inulinase promotes fructan hydrolysis under simulated gastric conditions

**DOI:** 10.3389/fnut.2023.1129329

**Published:** 2023-05-23

**Authors:** Justin L. Guice, Morgan D. Hollins, James G. Farmar, Kelly M. Tinker, Sean M. Garvey

**Affiliations:** Department of Research and Development, BIO-CAT, Inc., Troy, VA, United States

**Keywords:** digestion, enzyme, FODMAP, fructan, INFOGEST, inulin, inulinase, irritable bowel syndrome

## Abstract

Fermentable oligo-, di-, monosaccharides and polyols (FODMAPs) have emerged as key contributors to digestive discomfort and intolerance to certain vegetables, fruits, and plant-based foods. Although strategies exist to minimize FODMAP consumption and exposure, exogenous enzyme supplementation targeting the fructan-type FODMAPs has been underexploited. The objective of this study was to test the hydrolytic efficacy of a food-grade, non-genetically engineered microbial inulinase preparation toward inulin-type fructans in the INFOGEST *in vitro* static simulation of gastrointestinal (GI) digestion. Purified inulin was shown to undergo acid-mediated hydrolysis at high gastric acidity as well as predominantly inulinase-mediated hydrolysis at lower gastric acidity. Inulinase dose-response simulations of inulin, garlic, and high-fructan meal digestion in the gastric phase suggest that as little as 50 inulinase units (INU) and up to 800 INU per serving promote fructan hydrolysis better than the control simulations without inulinase. Liquid chromatography-mass spectrometry (LC-MS) profiling of fructo-oligosaccharides (FOS) in the gastric digestas following inulinase treatment confirms the fructolytic activity of inulinase under simulated digestive conditions. Altogether, these *in vitro* digestion data support the use of microbial inulinase as an exogenous enzyme supplement for reducing dietary fructan-type FODMAP exposure.

## 1. Introduction

Dietary fibers are carbohydrate polymers with ten or more monomeric units that are not digestible by endogenous enzymes in the human small intestine (1). While dietary fibers such as fructans, galactans, xylans, celluloses, and resistant starches are recalcitrant to human digestion, they are variably accessible to and fermented by different species and strains of the gut microbiota (2). Some dietary fibers are clinically substantiated prebiotics, which are defined as “a substrate that is selectively utilized by host microorganisms conferring a health benefit” (3). Beyond promoting the growth of beneficial gut microbes, dietary intake of certain digestion-resistant, fermentable prebiotics is associated with reduced risk for cardiovascular disease, type 2 diabetes, and some cancers (3–6).

Being polymers, dietary fibers can come in many sizes. The subject of this study, fructans [or fructooligosaccharides (FOS)], are polymers of fructose anchored to a single terminal glucose. The short-chain fructans, kestose (Glu-Fru_2_), nystose (Glu-Fru_3_), fructosylnystose (Glu-Fru_4_), and so forth, differ functionally and categorically by degree of polymerization (DP), or the number of total hexose residues. Fructans commonly found in garlic, onions, wheat, and bananas, possess a DP < 10 (7). The prebiotic inulin, by contrast, is a long-chain fructan (Glu-Fru_*n*_) with average DP > 10. Inulin is produced by several plants, such as Jerusalem artichoke (DP < 40), chicory root (DP < 100), and agave (8). Several clinical trials of inulin supplementation have demonstrated its utility as a prebiotic (4). Systematic review and meta-analyses of qualifying clinical trials have convincingly showed that oral supplementation of inulin or inulin-type fructans promotes prebiotic effects, such as increased fecal abundance of *Bifidobacterium* and *Lactobacillus* spp., improve blood lipid profiles, and improved mineral absorption (4, 9).

Despite numerous health benefits, dietary fiber can also lead to gastrointestinal (GI) distress, including symptoms such as abdominal bloating, abdominal pain, flatulence, diarrhea, and constipation (10, 11). These symptoms can arise from the fermentation of dietary fiber by microbes in the colon. Partly due to such symptomology, compliance with dietary fiber intake recommendations has been low in the US (6). In the past decade, dietary fermentable oligo-, di-, monosaccharides and polyols (FODMAPs) have emerged as key contributors to increased GI symptoms, food intolerance, and food sensitivity toward certain fruits, vegetables, beans, grains, and plant-based foods among both healthy individuals and individuals with irritable bowel syndrome (IBS). Food intolerance and sensitivity occurs through non-immune-mediated mechanisms that are distinct from food allergies (12–14). While FODMAPs include a broad range of carbohydrates, it is the fructans and galactans [or galactooligosaccharides (GOS)] that are particularly aggravating with respect to food intolerance. As little as 5 g FOS/day in healthy adults and 2 g FOS/day in individuals with IBS was sufficient to evoke increased GI symptoms (15, 16). Although these GI symptoms occur mildly in healthy individuals, individuals with IBS are more likely to suffer severe symptoms such as abdominal bloating and abdominal pain (17, 18).

Several dietary strategies have been developed to minimize the impact of FODMAPs and fructose on the GI system. One approach aims to modulate FODMAP consumption by upstream processing of ingredients and foods to reduce their FODMAP content (19, 20). Fractionation and isolation of FODMAP ingredients, carbohydrate leaching through cooking, and enzyme-mediated hydrolysis have also proved successful in reducing FOS, GOS, and excess fructose content in foods (21–25). In a second approach, low FODMAP diets are recommended to intolerant individuals to reduce intestinal microbial fermentation and related GI symptoms, followed by the gradual reintroduction of FODMAP foods to identify particularly aggravating foods to avoid (26). Low FODMAP diets in individuals with IBS have been shown to effectively reduce GI symptoms in clinical trials (17, 27–30). However, O’Keeffe et al. found that the majority of participants who reported symptom relief with FODMAP restriction also noted increased monetary food costs and difficulty finding appropriate foods at social eating occasions (16). The low FODMAP diet also carries a burdensome requirement for implementation by a trained dietetic specialist (31).

Another approach to limit net FODMAP exposure is exogenous enzyme supplementation. Supplemental enzymes have the potential to reduce FODMAP burden through enhanced gastric and intestinal digestion without restricting healthy, fiber-rich food choices. For example, alpha-galactosidase facilitates the successive cleavage of terminal galactose residues from GOS (32). Oral supplementation with alpha-galactosidase obtained from *Aspergillus niger* (the active ingredient in beano^®^) has been shown in multiple clinical trials to reduce bloating, flatulence, gas production, and gas-related symptoms associated with intolerance to legumes and other high-fiber, FODMAP-containing foods (33–36). This approach was also tested in individuals with IBS, successfully reducing GI symptoms in a study focusing solely on a GOS challenge and co-administration of alpha-galactosidase (37).

Despite the efficacy of microbial alpha-galactosidase for reducing net galactan and GOS exposure, there remains an opportunity to target fructan and FOS exposure. One solution is microbial inulinase. Inulinase is a β-fructofuranosidase, or fructan hydrolase, that hydrolyzes the 2,1-linked β-glycosidic bonds between fructose monomers (Fru–Fru), and between fructose and the terminal sucrose (Glu–Fru) of fructans such as inulin. For decades, inulinase obtained from bacterial (*Bacillus* sp., *Xanthomonas* sp., *Streptomyces* sp., etc.), yeast (*Kluyveromyces* sp., *Cryptococcus* sp., *Pichia* sp., etc.), and other fungal sources (*Aspergillus* sp., *Rhizopus* sp., *Penicillium* sp., *Rhizoctonia* sp., etc.), have been used for various industrial processes (38). In plants and microorganisms, inulinases break down fructans to release fructose for metabolic energy. Inulinases can exhibit exo-inulinase activity, endo-inulinase activity, beta-glucosidase activity, as well as invertase activity. While inulinase and invertase both catalyze the hydrolysis of FOS-rich inulins and sucrose, inulinase has a higher specificity for inulin than invertase, and invertase typically shows greater specificity for sucrose (39). Enzyme-mediated hydrolysis of inulin has been used to produce biofuels, fructose syrups, citric acid, lactic acid, and sugar alcohols (40). Similar to alpha-galactosidase, inulinase may also have value as a dietary supplement to promote greater GI tolerance to FODMAP containing foods. The aim of the present study was to investigate the efficacy of a food-grade, microbial inulinase preparation with companion invertase activity on several dietary fructan-rich substrates in the static INFOGEST *in vitro* digestion simulation.

## 2. Materials and methods

### 2.1. Enzymes

An inulinase preparation from wild-type, non-genetically engineered *Aspergillus tubingensis* (formerly *A. niger*) was used throughout all experimentation (Product Name: Inulinase, BIO-CAT, Inc.; Troy, VA, USA). BIO-CAT Inulinase [Chemical Abstract Service (CAS) Registry No. 37288-56-5] is an enzyme preparation containing a fermentation-derived filtered extract and tapioca maltodextrin at an approximate 3:7 ratio. Mass spectrophotometric analysis has confirmed that BIO-CAT Inulinase contains peptides mapping to *A. niger* extracellular exo-inulinase [UniProt Knowledgebase Accession (AC) No. A2R0E0, Enzyme Commission (EC) No. 3.2.1.80], as well as other carbohydrases such as β-fructofuranosidase (or invertase, AC No. A2QSK6, EC No. 3.2.1.26) and a putative β-glucosidase A (AC No. A2RAL4, EC No. 3.2.1.21, unpublished data). BIO-CAT Inulinase is standardized at no less than 2,000 inulinase activity units (INU) per gram. Characterization and expression of inulinase genes from microbial and fungal sources, including *Aspergillus* spp., have previously been described (41).

### 2.2. Test substrates

Substrates for inulinase testing inculded: (i) Orafti^®^ GR inulin (BENEO GmbH; Mannheim, Germany), hereafter referred to as inulin, (ii) garlic (minced garlic, Spice World, Inc.; Orlando, FL, USA), (iii) a standardized canned test meal (CTM) spiked with inulin (iCTM), (iv) CTM spiked with garlic (gCTM), and (v) a high FODMAP test meal (FTM). Briefly, the CTM contains 105 g canned chicken, 200 g mashed potatoes, 140 g drained green peas, and 23 g unsalted butter. The nutrient profile of the CTM, is 461 kcal, 45 g carbohydrate (48%), 31 g protein (33%), 17 g fat (19%), 11 g dietary fiber and has been previously described in detail (42). The iCTM substrate was prepared by adding 0.3 g inulin dissolved in 4.7 ml deionized water to 5 g CTM. The gCTM substrate was prepared by adding 5 g drained garlic to 5 g CTM. A second inulin, “inulin from chicory” (Product No. I2255, CAS No. 9005-80-5, Sigma-Aldrich; St. Louis, MO, USA) that is used in the inulinase activity assay, was also tested and is described in Supplementary Figures 2, 3.

The FTM was designed to contain an appropriate balance of macronutrients and a high level of FOS. The FTM comprises a black bean patty, Brussels sprouts, and sautéed garlic and onions. To prepare the black bean patty, 1 can of black beans (Bush’s Black Beans, Bush Brothers & Company; Knoxville, TN, USA) was drained and rinsed. Black beans (260 g) were baked in an oven on parchment paper at 177°C for 10 min. Separately, 60 g green bell pepper and 115 g white onion were minced in a food processor. Black beans were mashed in a bowl, and combined with the green pepper and onion mixture, 15 g garlic powder, 25 g onion powder, 6 g chili powder, 2.5 g cumin, and 1.5 g salt. An egg-like binder was made with 8 g flaxseed meal (Golden Flaxseed meal, Bob’s Red Mill Natural Foods, Inc.; Milwaukie, OR, USA) combined with 40 ml deionized water. Gluten-free bread crumbs (55 g, plain gluten free bread crumbs, 4C Foods Corporation; Brooklyn, NY, USA) and the flaxseed binder were added to the black bean mixture. The ingredients were mixed by hand and were weighed and shaped into 100 g patties. Black bean patties were baked on a lightly oiled (extra virgin olive oil, California Olive Ranch; Chico, CA, USA) baking sheet at 177°C for 10 min on each side. Brussels sprouts were trimmed at the base and 45 g sprouts steamed in a hanging basket over one inch of boiling water for 7 min. White onions were cut into wedges and 5 g garlic cloves were finely minced. In a non-stick pan, 46 g onion wedges were sautéed in 5 g extra virgin olive oil until onions began to lightly brown. The freshly prepared minced garlic was added to the onions prior to browning. The final FTM preparation contains 100 g black bean patty (uncooked weight), 45 g steamed Brussels sprouts, 46 g sautéed onions, 5 g sautéed garlic, and 5 g oil. Proximate analysis of 3 independent FTM preparations demonstrated the following average nutrient content per 201.0 g: 285 kcal, 45 g carbohydrate (73%), 10 g protein (16%), 7 g fat (11%), 334 mg sodium (15% daily value). Two randomly selected FTM preparations were further analyzed for fiber content, and indicated an average dietary fiber content of 12 g. Total FODMAP content from contributing ingredients (black beans, onion, garlic, and Brussels sprouts) was estimated to be 10 g per FTM. No direct quantitation of FODMAP content was performed. Details of the FTM composition, proximate analyses, and a photograph are included in Supplementary Table 1 and Supplementary Figure 1.

### 2.3. Inulinase activity assay

#### 2.3.1. Reagents and solutions

The substrate solution was prepared by dissolving 0.56 g inulin (Sigma-Aldrich) in 70 ml deionized water and heating in a boiling water bath for 3 min. After cooling to room temperature, 10 ml 1M acetic acid was added to the substrate solution, and the pH was adjusted to 4.5 ± 0.05 with 1M sodium hydroxide. The substrate solution was then transferred to a volumetric flask and diluted to 100 ml with deionized water. A 3,5-dinitrosalicylic acid (DNS, Product No. D0550, CAS No. 609-99-4, Sigma-Aldrich) solution was prepared in a 1 L beaker using 10 g DNS suspended in 400 ml warm deionized water, and kept below 60°C. Separately, 16 g sodium hydroxide was dissolved in 150 ml deionized water and slowly added to the DNS solution. After warming the solution to 50°C, 300 g sodium potassium tartrate was slowly combined with the DNS solution under continuous agitation and diluted to 1,900 ml with deionized water. The DNS solution was then diluted to 1 L after cooling to room temperature. DNS solution was stored at room temperature in an amber bottle in dark conditions and filtered through a sterile 0.22 μm PES membrane filter before use. A lactose solution was prepared by dissolving 1.2 g β-lactose (Product No. L3750, CAS No. 5965-66-2, Sigma-Aldrich; St. Louis, MO, USA) with 80 ml deionized water in a 100 ml volumetric flask, and diluted to 100 ml. A 1:100 dilution was prepared from the lactose stock solution for use in the assay. A DNS-lactose solution was freshly prepared by mixing 150 ml DNS solution with 50 mL diluted lactose solution. Fructose standards were prepared from a stock solution of 1.25 g D-fructose (Product No. F0127, CAS No. 57-48-7, Sigma-Aldrich; St. Louis, MO, USA) diluted to volume in a 50 mL volumetric flask. Three standards were prepared from diluted fructose stock to contain 0.10/0.2 ml, 0.20/0.2 ml, and 0.30/0.2 mL fructose. Separately, inulinase samples were prepared by diluting inulinase in deionized water to match an absorbance range of 0.15–0.40 in an ultraviolet–visible spectrophotometer set at 540 nm.

#### 2.3.2. Inulinase activity assay

The inulinase activity assay is based on a Miller reaction wherein fructose produced by inulin hydrolysis reduces 3,5-dinitrosalicylic acid (DNS) to 3-amino-5-nitrosalicylic acid, which can be detected photometrically. In an 18 × 130 mm test tube, 1.8 ml substrate solution was warmed 40 ± 0.5°C. The enzyme reaction began by mixing 0.2 ml inulinase sample with the substrate solution and was left to equilibrate at 40 ± 0.5°C for 20 min. To stop the reaction, 4 ml DNS-lactose solution was added and the mixture placed, covered, into a boiling water bath for 15 min. Inulinase reaction samples were cooled to room temperature in a cooling water bath and absorbance determined at 540 nm. A reaction blank was prepared by combining 0.2 ml inulinase sample solution, 4 ml DNS-lactose solution, and 1.8 ml substrate solution in a test tube. Tubes were covered with plastic stoppers and placed in a boiling water bath for 15 min. The reaction blank was then cooled to room temperature and absorbance determined at 540 nm using deionized water to blank the spectrophotometer. Fructose standard dilutions and a water blank were similarly prepared. Here, 0.2 ml fructose standard or deionized water were pipetted into test tubes. Then, 1.8 ml substrate solution and 4 ml DNS-lactose solution was added to the test tubes and were mixed in succession. Tubes were covered with plastic stoppers and placed in a boiling water bath for 15 min. The fructose and water blanks were then cooled to room temperature and absorbance determined at 540 nm. One inulinase unit (INU) is defined as the quantity of enzyme needed to liberate reducing sugars, such as fructose, at a rate of 1 μmol per minute at pH 4.5 and 40°C. Inulinase activity is defined:


$$\mathrm{Inulinase}\,\mathrm{Activity}\,\left(u/g\right)=\left({\text{A}}_{\text{T}}-{\text{A}}_{\text{B}}\right)\times F\times \frac{1,000}{180}$$

$$\times \frac{0.2\,\mathrm{mL}}{20\,\min}\times \frac{1}{0.2\,\mathrm{mL}}\times \frac{1}{\text{W}}$$

where: A_*T*_, absorbance of the enzyme reaction solution; A_*B*_, absorbance of the reaction blank; F, fructose factor (mg/m); 1,000, mg to μg conversion; 180, molecular weight of fructose; 0.2 ml, fructose standard solution volume to test; 20 min, reaction time; 0.2 ml, inulinase sample solution volume to test; W, weight, in grams, of the enzyme preparation contained in the 1.0 ml of the diluted sample preparation; and the fructose factor (F) is defined as:


$$\text{F}=\frac{{\text{C}}_{\text{F}}}{{\text{A}}_{\text{F}}-{\text{A}}_{\text{W}}}$$

where: C_*F*_, concentration of fructose standard dilution (mg/ml), A_*F*_, absorbance of fructose standard dilution; A_*w*_, absorbance of water blank.

**FIGURE 1:**
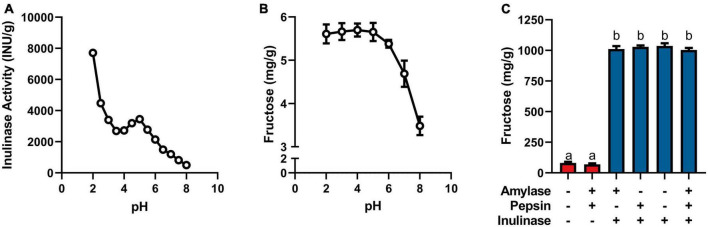
**(A)** Inulinase activity across usual human GI pH using a modified, industry-standard inulinase activity assay. **(B)** Fructose concentration of the inulinase assay reaction solution across usual human GI pH. **(C)** Fructose concentration of gastric digestas following simulated digestion of inulin with and without inclusion of inulinase or porcine amylase and pepsin. Error bars show ± 1 standard deviation. Significant differences (*p* < 0.05) between samples are denoted by unshared lower-case letters (a, b).

This standard inulinase assay was used to determine doses of inulinase based on INU, rather than weight alone, so that results can be compared across studies and laboratories. The pH of the inulinase assay was modified in one set of experiments from the standard pH 4.5 to a series of pH values that span the human GI pH range (see Figure 1).

### 2.4. Digestive enzyme assays

The assays used to measure the activity of each lot of porcine salivary amylase and porcine pepsin have previously been described (42–44).

### 2.5. Blender protocol

A Hamilton Beach Commercial HBB908 bar blender (Hamilton Beach Brands, Inc.; Glen Allen, VA, USA) was used to generate a mash of the test meals to approximate the consistency of masticated food. When blending was not performed immediately after cooking, food was plated on a Corning Ware porcelain dinner plate and stored at 4°C for up to 2 days. Food was microwaved until warm and no more than 90 s. In the assembled blender bowl, the cooked food was combined with 100 ml of deionized water. The blades were initially pulsed 2–3 times to redistribute food, then continuously blended in the vessel until the test meal showed a uniform consistency. This test meal mash was divided into 10 g aliquots and stored in a tightly capped container (Nalgene™ Straight-Side Polycarbonate Jars with Caps; Thermo Fisher Scientific, Waltham, MA, USA) at −20°C for up to 30 days.

### 2.6. *In vitro* digestion with the INFOGEST static model

The INFOGEST consensus method describes a static GI simulation of digestion. The full INFOGEST protocol (45, 46) and its adaptation to the study of exogenous enzymes (42), have previously been described. Briefly, the protocol is used to model three phases of digestion: salivary, gastric, and intestinal. The salivary phase combines the food sample with a simulated salivary fluid containing porcine salivary amylase under agitation at neutral pH and 37°C for 2 min. The gastric phase immediately follows the salivary phase with the addition of a simulated gastric fluid containing porcine pepsin at a starting pH 3.0 followed by agitation at 37°C for 2 hours. In the treatment groups, a partial dose of inulinase, based on the partial serving sizes of the starting substrates, was added to the gastric digesta 10 min into the gastric phase to model dissolution of a dietary supplement capsule shell. A subsequent intestinal phase proceeds by addition of a simulated intestinal fluid containing porcine pancreatin and bile salts at a starting pH 7.0 followed by agitation at 37°C for 2 h. Enzyme activity is terminated by boiling digestas at 90°C for 10 min.

In addition to standard static INFOGEST simulation, the effects of varying pH and absence of endogenous digestive enzymes on inulinase activity were also tested. Fructose concentration of digestas was measured after inulin was digested: (i) without amylase, pepsin and inulinase, (ii) with amylase and pepsin, but without inulinase (iii) without pepsin, with amylase and inulinase (400 INU/serving), (iv) without amylase, with pepsin and inulinase, (v) without amylase and pepsin, and with inulinase, and (vi) with amylase, pepsin, and inulinase. To further test the efficacy of inulinase, dose-response experiments were performed with inulin, garlic, iCTM, gCTM, and FTM. Doses doubled in activity from 3.125 to 800 INU/serving for inulin and garlic, and 50 to 800 INU/serving for iCTM, gCTM, and FTM. Efficacy was based on increased digesta free fructose concentration compared to controls with endogenous enzymes alone.

### 2.7. Quantitation of sugars by HPLC

Gastric or intestinal digestas were vortexed and filtered through a 0.45 μm nylon syringe filter prior to dilution in deionized water for evaluation of fructan hydrolysis by high-performance liquid chromatography (HPLC) with refractive index detection (Agilent 1100 Series, Agilent Technologies, Inc.; Santa Clara, CA, USA). Fructose from the gastric or intestinal digesta was analyzed on an HPLC with a SUPELCOGEL C-610H 30 cm × 7.8 mm column at 30°C and a flow rate of 0.5 ml/min of 0.1% phosphoric acid. A 5-point calibration curve was generated from fructose standards (mg/ml): 0.05, 0.1, 0.25, 0.5, 0.75, 1. Results are reported in mg fructose/g starting substrate (wet weight).

### 2.8. Fructan analysis by LC-MS

Each experimental digesta was analyzed with a liquid chromatography–mass spectrometry (LC-MS) system using a previously published method (47). The system consisted of an Agilent G6545A QToF Mass Spectrometer with a Dual Agilent Jet Stream Electrospray Ionization interface and an Agilent 1260 Infinity II LC System with a quaternary pump, autosampler, and column manager (Agilent Technologies, Inc.; Santa Clara, CA, USA). The column was a 4.6 × 50 mm YMC-Pack ODS-AQ with a 3 μm particle size (PN AQ12S03-0546WT).

Inulin, garlic, and FTM digestas from the INFOGEST digestion were profiled for inulinase efficacy and characterization of remaining fructans. The digestas were diluted into 0.01% formic acid (1:9), filtered (0.2 μm), and injected into the LC-MS for a nominal load of ∼1 μg of total FOS. Chromatographic separation was performed with a binary gradient of mobile phases A [water/1% formic acid (990/10 v/v)) and B (acetonitrile/1% aqueous formic acid (990/10 v/v)]. The gradient (min/%B/Flow) included 0.0/0/600; 5.0/0/700; 40.0/4/700; 45.0/80/700; 46.0/80/700; 47.0/0/700; 57.0/0/700; and 58.0/0/600 parameters and was held at 30^°^C for each run. All reagents and solvents were LC-MS grade or the highest purity available.

The mass spectrometer was run in positive mode using the following parameters: gas temperature: 325°C; gas flow: 12 L/min; nebulizer: 40 psi; sheath gas temperature: 350°C; sheath gas flow: 12 L/min; capillary voltage: 3,500 V; nozzle voltage: 500 V; fragmentor: 175 V; skimmer: 60V; octopole RF peak: 750 V; mass range: 100–3,000 m/z; scan rate: 2 spectra/s; 500 ms/spectrum. The internal reference standards (G1969-85003, Agilent, Santa Clara, CA, USA) generated calibrant ions of 121.0509 and 1,821.9523 m/z.

Identification and quantitation of oligosaccharides were performed using the Target/Suspect Screening workflow application (Agilent MassHunter Qualitative Analysis v10.0). The application used a database of chemical formulas corresponding to distinctly sized inulooligosaccharides (IOS) (based on DP), created with Agilent MassHunter PCDL Manager (Supplementary Table 2). Note that the chemical formulas would match any hexose sugar present in the digesta, not just IOS. Confirmation of DP was dependent upon matching the predicted ions with the IOS database (Supplementary Table 2) or with absence of the peak following inulinase treatment. Additionally, inulins DP5 and DP8 eluted much later than maltopentaose (Glu_5_, DP5), maltooctaose (Glu_8_, DP8), stachyose (Gal_2_-Glu-Fru, DP4) and maltodextrin (Glu_*n*_, DPs 2–17) (data not shown). To enable detection of isomers for any DP, retention times were not assigned to chemical formulas in the database. The sodium adduct was the predominant ion for each inulin DP. The software produced an extracted ion chromatogram (EIC) for each charge state of each inulin DP using a mass tolerance window of 10 ppm. The areas under the curve for each DP charge state were summed into a single peak for each inulin DP. Those EICs which had a score greater than 60 were included. The score was based on the mass, isotope abundance and isotope spacing of each EIC. All of the confirmed inulin DPs were overlaid into a single chromatogram to show the relative amounts of inulin DPs detected. LC-MS analyses were performed on control, 50 INU/serving, and 400 INU/serving digestas and overlaid EICs were subsequently generated.

### 2.9. Statistical analysis

A one-way ANOVA was performed separately for the dose-response experiments (inulin, garlic, iCTM, gCTM, FTM). To account for multiple comparisons, a Tukey HSD was performed post-hoc. Normality was assessed by the Shapiro–Wilk test on residuals and visually by QQ-plot. Homoscedasticity was confirmed using the Brown-Forsythe test and homoscedasticity plot. The significance level was set at 5% (*p* ≤ 0.05) for all analyses. Statistical analyses were performed and figures were generated in GraphPad Prism version 9.2.0 for Windows (GraphPad Software, Inc.; San Diego, CA, USA).

## 3. Results

### 3.1. Effects of pH on inulin hydrolysis and inulinase activity

Inulinase demonstrated fructolytic activity under industry-standard, inulinase activity assay conditions at pH 4.5 (Figure 1A). In this assay, inulin (Sigma-Aldrich) is prescribed as the substrate, and fructose release is indirectly measured by spectrophotometric detection of a product of its reaction with DNS. The assay was modified beyond pH 4.5 to span the usual human GI pH range (Figure 1A). The pH × enzyme activity curve showed highest inulinase activity below pH 3, decreasing to a nadir at pH 3.5, peaking again at pH 5, and decreasing nearly to extinction at pH 8 (Figure 1A). Acid hydrolysis was likely contributing to the apparent peak in inulinase activity at lower pH, while the second peak around pH 5 was due solely to inulinase activity. Acid-mediated hydrolysis of inulin at pH < 4 has previously been described (8). Additionally, we directly measured fructose concentration by HPLC at the end of the pH-modified inulinase activity assay. Fructose serves as a more direct measure of fructan hydrolysis and is expressed as mg fructose per g inulin substrate (wet weight). The pH × concentration curve showed no difference in peak fructose concentrations from pH 2–6, at which pH inulin oligosaccharides may be fully hydrolyzed to fructose by inulinase, acid, or a combination thereof (Figure 1B). Above pH 6, fructose concentrations were reduced by 18 and 39% at pH 7 and 8, respectively, compared to the maximum concentration at pH 4 (Figure 1B). The discrepancy in relative efficacy at pH 3.5 and 4.5 between the DNS-based outcome and the free fructose concentration may be attributable to minor endo-inulinase activity and the presence of short-chain FOS at pH 4.5. There may also be an interfering artifact effect with the use of DNS at certain pH, however more experimentation is needed.

Endogenous digestive enzymes had no effect on inulinase activity during simulated gastric digestion of inulin (*p* < 0.0001, Figure 1C). Without inulinase, amylase and pepsin inclusion showed no difference in fructose concentration compared to a control simulation without any enzymes present (Figure 1C). With inulinase treatment alone (400 INU/serving), the enhanced fructose concentration was no different than inulinase treatment in the presence of amylase, pepsin, or both (Figure 1C).

### 3.2. Simulated digestion of fructans by inulinase

**FIGURE 2:**
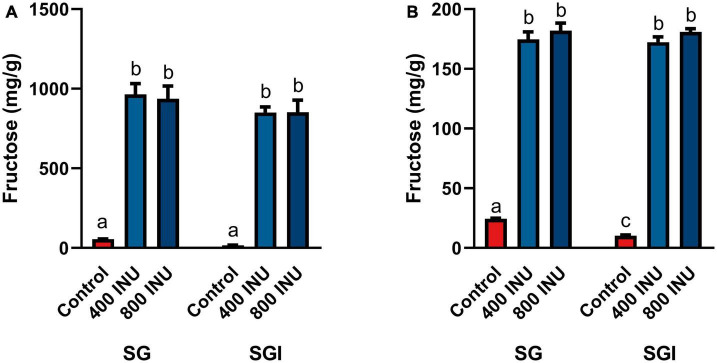
Fructose concentrations of digestas after inulinase treatment of **(A)** inulin and **(B)** garlic under standard INFOGEST salivary-gastric (SG) and salivary-gastric-intestinal (SGI) conditions (*n* = 3). Error bars show ± 1 standard deviation. Significant differences (*p* < 0.05) between samples are denoted by unshared lower-case letters (a, b, c). INU, inulinase activity unit per serving.

The *in vitro* static INFOGEST simulation of gastrointestinal digestion was used to investigate the effects of inulinase on fructan hydrolysis. Efficacy was based on the concentration of fructose in the digestas following simulated digestion, since dietary fructan hydrolysis increases the amount of free fructose. In these experiments, inulinase activities were reported in inulinase units per approximately one serving size of inulin, garlic, or mixed meal. The full salivary-gastric-intestinal (SGI) simulation of each of inulin and garlic digestion was compared to only the salivary-gastric (SG) phases to assess any incremental benefit of inulinase treatment through the intestinal phase. In the SG simulations of inulin and garlic digestion, each of 400 INU/serving and 800 INU/serving doses of inulinase led to significantly elevated free fructose concentrations compared to the control group with salivary amylase and pepsin alone. In the SGI simulation, inulinase treatment also increased fructose concentrations of the intestinal digestas, however no different from the gastric digestas (400 and 800 INU/serving, inulin, *p* > 0.1000; garlic, *p* > 0.9000; Figures 2A, B). Since the majority of fructan hydrolysis occurred during the gastric phase, all remaining experiments were carried out with the SG simulation without the intestinal phase.

**FIGURE 3:**
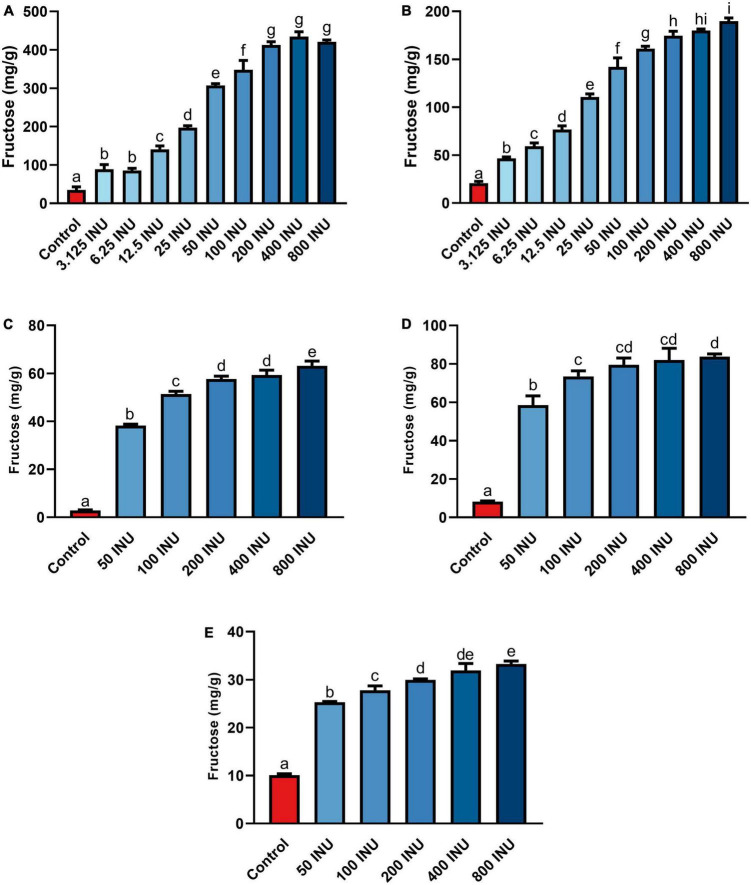
Fructose concentrations of gastric digestas after inulinase treatment of **(A)** inulin, **(B)** garlic, **(C)** iCTM, **(D)** gCTM, and **(E)** FTM under standard INFOGEST salivary-gastric conditions (*n* = 3 for all series of experiments). Error bars show ± 1 standard deviation. Significant differences (*p* < 0.05) between treatments are denoted by unshared lower-case letters (a, b, c, d, e, f, g, h, or i) for each series. INU, inulinase activity unit per serving.

Fructan-rich substrates included a purified inulin and increasingly complex substrates incorporating food mashes: garlic, an inulin-spiked canned test meal (iCTM), a garlic-spiked canned test meal (gCTM), and a high FODMAP test meal (FTM). Hydrolysis of purified inulin (BENEO Orafti^®^ GR) was examined first. Fructose concentration increased with all inulinase doses compared to control (*p* < 0.0001, Figure 3A and Supplementary Table 3). Fructose concentrations increased significantly between each dose from 6.25 to 50 INU (Supplementary Table 3). No differences between 3.125 and 6.25 INU, 50 and 100 INU, nor for doses above 200 INU were observed (Supplementary Table 3). Inulinase at 3.125 INU resulted in a 2.6-fold difference from control (88.9 mg/g vs. 34.8 mg/g fructose, *p* = 0.0003), whereas the largest difference from control was 12.5-fold greater at 400 INU (434.6 mg/g vs. 34.8 mg/g fructose, *p* < 0.0001) (Supplementary Table 3). Inulinase treatment of a second inulin source from Sigma-Aldrich, which is the prescribed substrate in the industry-standard inulinase activity assay, also led to increased gastric digesta fructose concentrations compared to control (*p* < 0.0001, Supplementary Figure 2)

**FIGURE 4:**
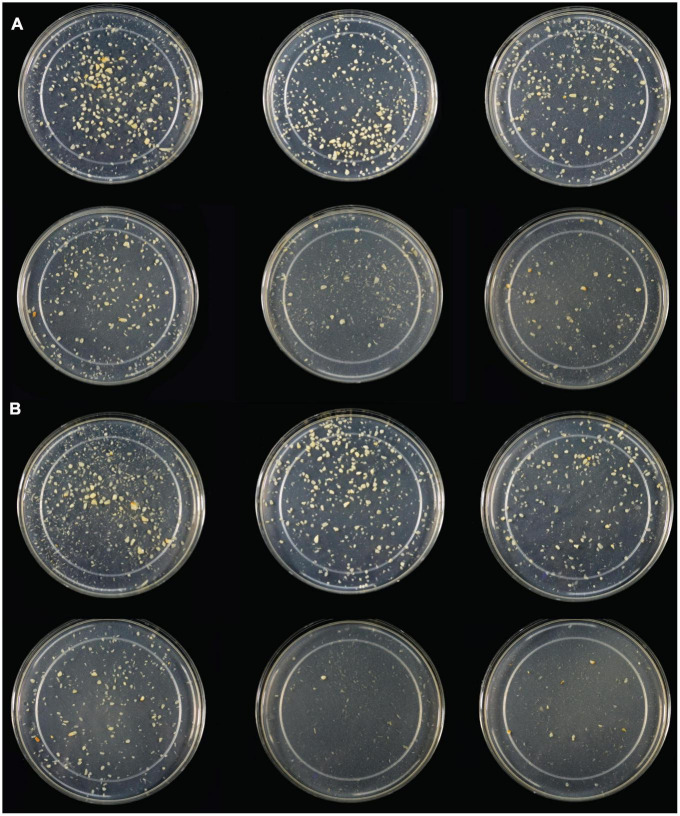
**(A)** Light photograph of gastric digestas after inulinase treatment of minced garlic under standard INFOGEST salivary-gastric conditions (2-h gastric phase); **(B)** Light photograph of gastric digestas after inulinase treatment of garlic under modified INFOGEST salivary-gastric conditions comprising a 4-h gastric phase; Left to right, top to bottom: Control, 50, 100, 200, 400, and 800 INU per serving.

Following SG simulation of garlic digestion, differences in fructose concentrations observed between inulinase treatments and control were statistically significant (*p* < 0.0001, Figure 3B and Supplementary Table 3). Inulinase treatment increased the fructose concentration 2.3-fold at the lowest tested activity (3.125 INU), compared to control (46.6 mg/g vs 20.7 mg/g fructose, *p* < 0.0001), and up to a 9.2-fold increase at the 800 INU dose (189.7 mg/g vs 20.7 mg/g fructose, *p* < 0.0001) (Supplementary Table 3). There were no significant differences between 200 INU and 400 INU, and 400 INU and 800 INU (Supplementary Table 3). Visual inspection of the gastric digestas following 2 hours of simulated SG digestion showed a dose-dependent reduction in the size of garlic fragments, with marked dissolution at the 400 and 800 INU doses (Figure 4A). In a modified protocol, duration of the gastric phase was doubled to 4 hours, and dissolution of the garlic fragments was nearly complete at the 400 and 800 INU doses by visual inspection, whereas the usual acidity of the standard INFOGEST protocol did not appear to appreciably reduce garlic fragment size under control conditions without inulinase (Figure 4B)

Inulin-spiked CTM (iCTM) was used to test inulinase efficacy on an established mixed meal matrix (42). Inulinase digested fructans from the iCTM similarly to purified inulin. The overall relationship did not change between the experiments (*p* < 0.0001, Figure 3C and Supplementary Table 3), and increased fructose concentrations were observed across all doses tested. Inulinase treatment increased the fructose concentration of the gastric digesta 13.6-fold at the 75 INU dose, compared to control (38.5 mg/g vs. 2.8 mg/g fructose, *p* < 0.0001), and up to a 22.4-fold increase (63.1 mg/g vs. 2.8 mg/g fructose, *p* < 0.0001) at the highest dose (Supplementary Table 3). The 200 and 400 INU doses did not differ (57.6 mg/g vs 59.3 mg/g fructose, *p* = 0.6719). Garlic-spiked CTM (gCTM) was also tested as a substrate, and fructose concentrations increased with inulinase treatment (*p* < 0.0001, Figure 3D and Supplementary Table 3). Gastric digestas following gCTM digestion with inulinase demonstrated a fructose concentration increase of 7.2-fold at the lowest inulinase dose, compared to control (58.5 mg/g vs. 8.2 mg/g fructose, *p* < 0.0001) and 10.3-fold at the highest dose (87.8 mg/g fructose, *p* < 0.0001) (Supplementary Table 3). Doses above 50 INU increased significantly compared to control, but were not substantially different from each other.

Inulinase efficacy was also tested on FTM—a mixed meal matrix whose fructan content is derived from several complex food sources including black beans, Brussels sprouts, onions, and garlic. There was a significant treatment effect of inulinase on FTM (Figure 3E and Supplementary Table 3). At the lowest inulinase dose, we observed a 2.5-fold fructose concentration increase compared to control (25.3 mg/g vs. 10.1 mg/g, *p* < 0.0001) and 3.3-fold increase at the highest dose (33.3 mg/g vs. 10.1 mg/g, *p* < 0.0001) dose (Supplementary Table 3).

**FIGURE 5:**
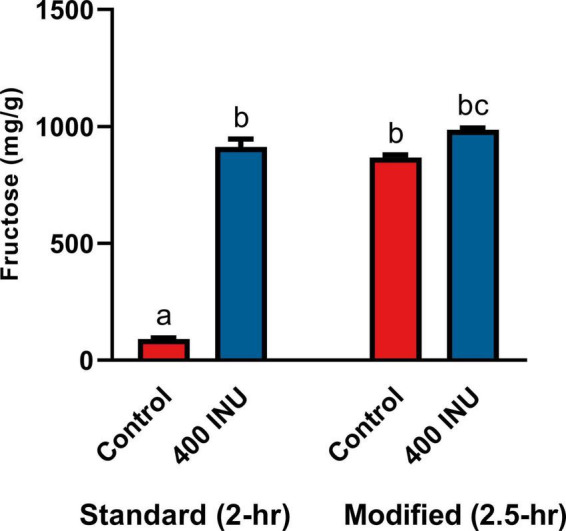
Fructose concentrations of gastric digestas after inulinase treatment of inulin under standard INFOGEST salivary-gastric conditions (*n* = 3) and modified conditions with an additional 30-min incubation with starting pH 2. Error bars show ± 1 standard deviation. Significant differences (*p* < 0.05) between samples are denoted by unshared lower-case letters (a, b, and c). INU, inulinase activity unit per serving.

The standard INFOGEST static digestion protocol includes a 2-hour gastric phase with a starting pH 3.0. A semi-dynamic INFOGEST gastric digestion protocol was recently described, wherein following a 100-min gastric phase, digestas were slowly acidified to pH 2 and then held constant at pH 2 for 50 additional minutes of incubation (48). The increased duration at higher acidity may be expected to better model digestion of larger meals with longer gastric emptying times. Between this report and the evidence for acid-mediated hydrolysis at pH 2 (Figure 1A), we performed an additional SG simulation of inulin digestion with an additional 30-min gastric sub-phase starting a pH 2. We observed a significantly increased fructose concentration in the control when held at pH 2 (867.0 mg/g vs. 90.6 mg/g, *p* = 0.0001, Figure 5), and a significant, but minor increase from standard INFOGEST conditions when treated with 400 INU inulinase (986.2 mg/g vs. 911.6 mg/g, *p* = 0.0056, Figure 5). These data suggest that inulin hydrolysis can be mediated by gastric acidity around pH 2, independent of supplemental inulinase.

### 3.3. Fructan profiling by LC-MS

**FIGURE 6:**
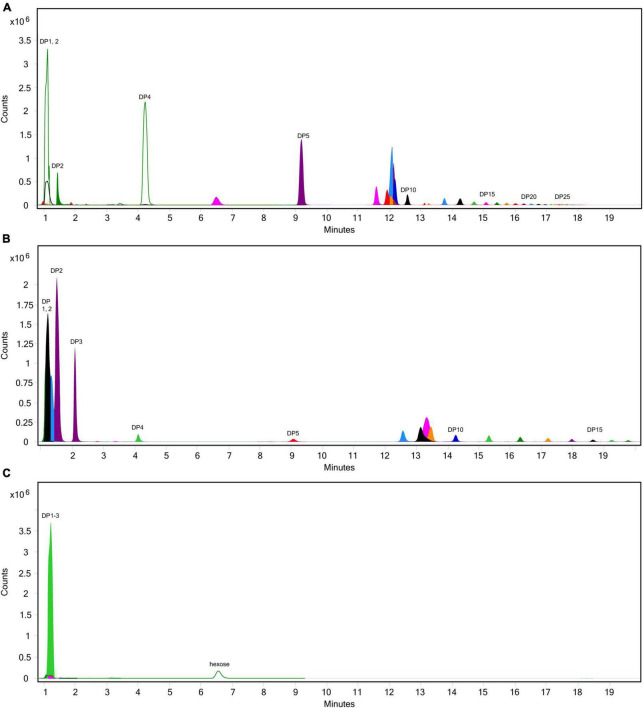
Overlaid extracted ion chromatograph of inulooligosaccharides (IOS) following simulated salivary-gastric digestion of inulin under standard INFOGEST conditions, **(A)** control, **(B)** 50 INU/serving, **(C)** 400 INU/serving.

**FIGURE 7:**
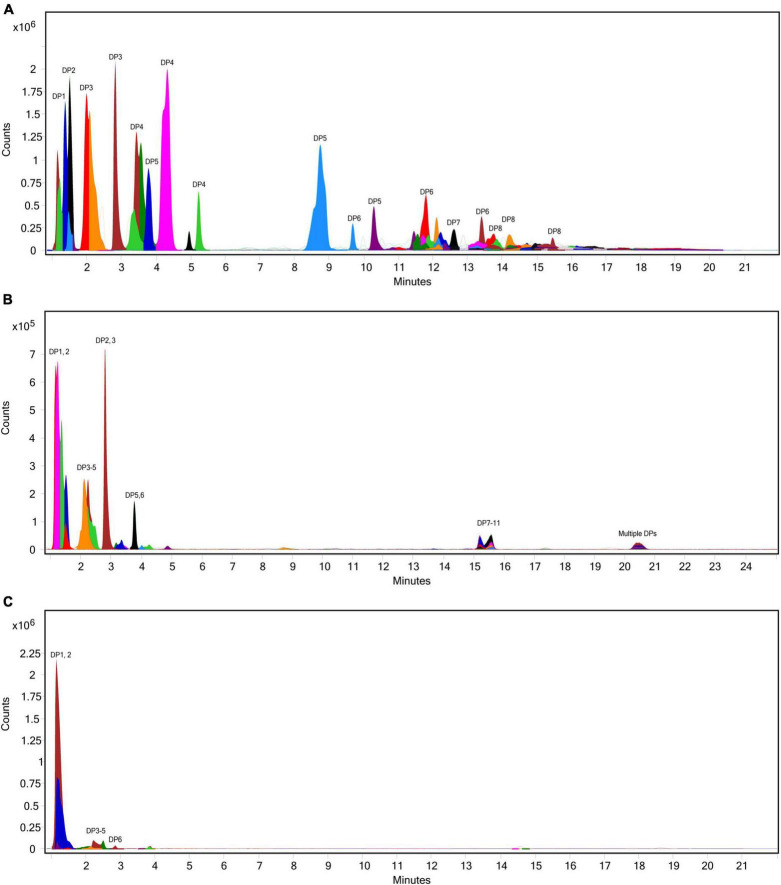
Overlaid extracted ion chromatograph of fructooligosaccharides (FOS) following simulated salivary-gastric digestion of garlic under standard INFOGEST conditions, **(A)** control, **(B)** 50 INU/serving, **(C)** 400 INU/serving.

**FIGURE 8:**
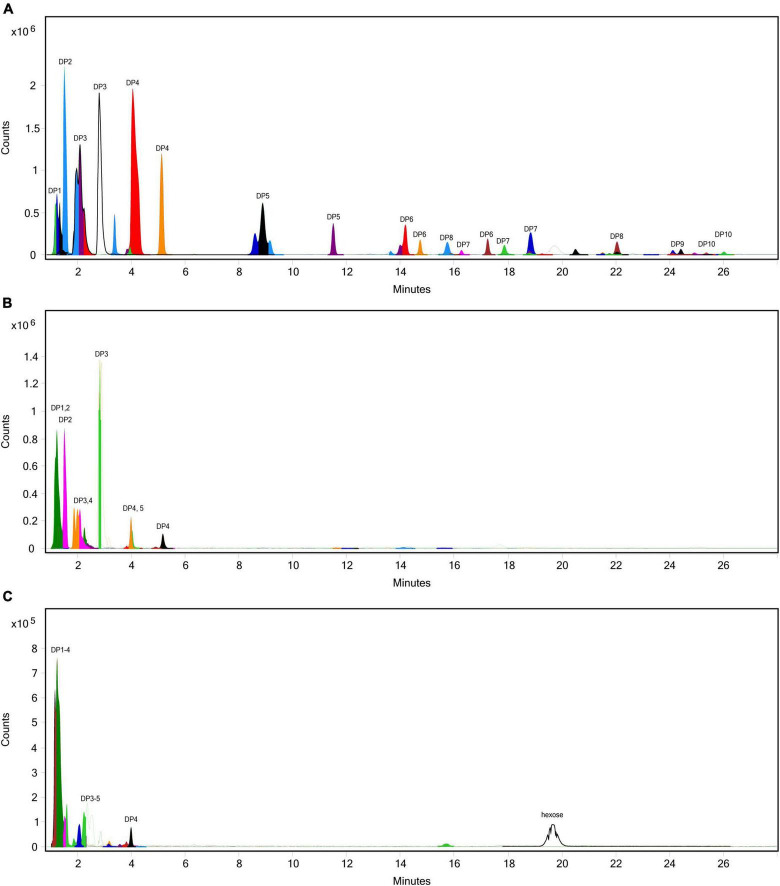
Overlaid extracted ion chromatograph of fructooligosaccharides (FOS) following simulated salivary-gastric digestion of the high FODMAP test meal (FTM) under standard INFOGEST conditions, **(A)** control, **(B)** 50 INU/serving, **(C)** 400 INU/serving.

The extent of fructan hydrolysis by inulinase was more precisely detailed by LC-MS. Gastric digestas from the simulations of inulin, garlic, and FTM digestion were examined for any remaining fructans. The inulin control digestas showed DPs 2–11 as the top 10 most abundant DPs with a steady decline in abundance out to DP34 (Figure 6A). Inulinase treatment at 50 INU reduced the concentration of fructans observed under control conditions (Figure 6B), while inulinase at 400 INU completely hydrolyzed all detectable greater than DP3 (Figure 6C). Simulated salivary-gastric digestion of a second inulin source—inulin from Sigma-Aldrich—yielded DPs 13–22 as the top 10 most abundant DPs with a Gaussian distribution centered on DP15 and a steady decline in abundance out to DP44 (Supplementary Figure 3). Control digestas for the garlic substrate showed a fructan range clustered around DPs 1–5 with multiple isomers as the top 10 most abundant DPs and a steady decline in abundance and isomers out to DP59 (Figure 7A). FTM digestas showed the lowest DP range of all tested substrates, likely a consequence of greater processing compared to pure inulin and uncooked, minced garlic. FTM control digestas contained DPs 2-6 with multiple isomers as the top 10 most abundant DPs and a steady decline in abundance and multiple DP isomers out to DP20 (Figure 8A). Similar to inulin, 50 INU showed significant digestion of all fructans greater than DP2 in garlic and FTM (Figures 7B, 8B). Inulinase at 400 INU completely hydrolyzed all fructans greater than DP2 in garlic and FTM (Figures 7C, 8C). Control digestas and inulinase treated digestas for iCTM and gCTM substrates (Supplementary Figures 4, 5) showed fructan profiles similar to inulin and garlic, respectively, suggesting the increased complexity of the substrate matrix does not affect inulinase activity.

## 4. Discussion

To our knowledge, this is the first published study focusing on the efficacy of inulinase alone—outside of a multi-enzyme supplement—in any type of GI digestion simulation. We showed that microbial inulinase is unaffected by endogenous salivary and gastric digestive enzymes and highly active within the acidic pH range of the human gastric compartment (Figure 1), as well as effective at digesting a variety of pure, simple, and complex fructan-rich substrates under simulated gastric conditions (Figure 3). Surprisingly, inulinase showed hydrolytic efficacy even at the lowest doses tested. Beyond HPLC analysis of fructose concentration, we employed LC-MS profiling to demonstrate nearly complete elimination of high-DP fructans from purified inulin and garlic with inulinase treatment (Figures 6–8). These data support inulinase stability and efficacy across physiologically relevant gastric conditions including both high acidity and the presence of pepsin, an endogenous gastric protease.

Exogenous supplementation with inulinase, fructanases, or fructose hydrolases may be useful for reducing the GI burden of FODMAPs in sensitive individuals and individuals with IBS. Such an approach was suggested by two patents dating back to the 1990s (49, 50), however, there remains no published clinical data regarding inulinase supplementation. Our *in vitro* data showing that inulinase catabolizes fructans at usual human gastric pH suggest that inulinase supplementation may be an effective solution for improving fructan intolerance. Less symptomatic food digestion with inulinase supplementation may also enable a healthier diet with greater plant-based, nutrient-rich, and fiber-rich food choices. Beyond explicit FODMAP intolerance, inulinase supplementation could also help facilitate a lifestyle transition toward a more plant-based, whole-food diet. When paired with additional glycan-targeting carbohydrases such as alpha-galactosidase and pectinase, we expect the digestive impact of FODMAPs to be even less pronounced. There may also be opportunity to incorporate inulinase supplementation into dietitian-supervised low FODMAP diet protocols. A clinical trial has been initiated to determine the safety and tolerability of inulinase supplementation (51). Overall, our *in vitro* data support effective doses in the range of 50–800 INU per serving. Although we did not quantitate the total concentration of fructans in each substrate, at lower inulinase doses, we expect a modest reduction of all fructans. For inulinase doses 400 INU per serving and greater, we expect long-chain fructans to be completely hydrolyzed, and short-chain fructans to be significantly, if not completely, degraded. Accordingly, inulinase hydrolyzed fructans associated with approximately 12 g dietary fiber per serving of FTM (Figure 3E). Future work will aim to better characterize fructan load in dietary substrates.

While inulinase functions across a broad pH range, we determined that inulinase activity was substantially reduced at intestinal pH (Figure 1). We found no differences in inulin hydrolysis when comparing gastric and intestinal phases, confirming that approximately neutral pH drove inulinase activity nearly to extinction. These observations align well with use of inulinase for food intolerance, since primary gastric activity would reduce the amount of fructan fermentation downstream in the colon, while also allowing the released fructose—itself a FODMAP—to be absorbed by the small intestine. In agreement with this proposed mechanism, it was recently reported that a multi-enzyme food supplement product containing inulinase activity digested inulin in a semi-dynamic digestion model concomitant to fructose absorption by a simulated intestinal epithelial barrier (52). For all these reasons, we focused on the elimination of fructans in the gastric phase. Nonetheless, future experimentation of inulinase with mixed meal substrates will incorporate a full GI simulation using semi-dynamic modeling. Independent of inulinase treatment, it is intriguing that inulin is hydrolyzed at pH 2, yet remains intact at pH ≥ 3 (Figure 5). These data suggest that there is likely interpersonal variability in the extent of inulin digestion based on usual gastric acidity and even gastric emptying time. An extreme example is the case of proton pump inhibitors (PPI) which effectively suppress gastric acidity in individuals with ulcers, reflux, and dyspepsia. Individuals who consume PPIs are expected to have gastric pH 1 to 3 units higher—well above pH 3—than usual gastric pH (53–55). Thus, PPI users are likely to be at greater risk of experiencing fructan intolerance. Since gastric acidity increases with longer gastric emptying times (lower pH), and larger meals typically lead to longer gastric emptying times (56), it is also predicted that fructans from a large meal would be less digestively aggravating than fructans from a snack such as a protein shake or nutritional bar. Since the upper pH limit of acid-mediated inulin hydrolysis may fall within the range of usual gastric acidity, even small changes in gastric emptying time or hydrogen ion output could impact fructan digestion and any related GI symptoms. These hypotheses will be important to test in future clinical research, as well as the impact of inulinase supplementation in reducing GI symptoms in those with FODMAP intolerance or higher than usual gastric acidity.

Beyond potentially reducing GI symptoms, inulinase-mediated fructan hydrolysis in the gastric compartment prior to intestinal fermentation may help support intestinal microbiota health. It is well established now from clinical trials that inulin is a prebiotic that promotes the growth of beneficial intestinal microbes (9, 57–61). However, an intestinal microbiota theoretically may be low or deficient in taxa with genes that support fructan metabolism and mediate the prebiotic effects of inulins. Exogenous inulinase supplementation could release more short-chain FOS to support the growth of beneficial microbes. A recent report by la Rosa et al. indicates use of glycans by the gut microbiome (62). Several other studies specifically demonstrate the utilization of glycans by *Bacteroidetes thetaiotaomicron* (63–65), signifying the prospect of inulinase supplementation to complement the gut microbiota in the production of volatile fatty acids. Interestingly, Sonnenburg et al. reported that *B. thetaiotaomicron* thrived poorly on inulin, but grew well on many fructose-containing carbohydrate medias, including free fructose, sucrose, FOS, and levan. Inulinase supplementation may enhance the microbiota by providing additional carbohydrates to supplant missing microbe-derived extracellular fructanase or stimulate secretion of fructanases normally produced by fructan degraders. Related, there is a renewed focus on the distinctions between the small intestinal microbiota, colonic, and fecal microbiota, and ultimately the importance of the duodenal microbiota (66). Beneficial duodenal microbes could benefit immediately from short-chain FOS released by inulinase in the stomach. Future studies will include digestion models that incorporate human fecal slurries to model the intestinal microbiota.

It may also become important to better understand the impact of distinct FOS molecules, as defined by DP, on prebiotic properties and inulin’s role as a dietary fiber. Through LC-MS profiling, it may be possible to identify DP ranges of inulins that differentially impact GI health. For example, it has been shown that, dependent upon DP, inulin fibers differentially modulate composition of the intestinal microbiota, protection against endotoxemia, and inflammation (67–69). Conversely, two weeks administration of a diet containing 30% fiber from inulin was shown to promote type 2 inflammation and exacerbate allergic symptoms in mice (70). In a separate study in mice, a high FODMAP diet was associated with intestinal mast cell activation and colonic epithelial barrier loss not observed in mice fed a low FODMAP diet (71). These findings were corroborated by another mouse study wherein oral administration of FOS—specifically—led to increased intestinal mast cell counts and dysregulation of the colonic epithelial barrier associated with production of advanced glycation end products in colonic epithelial cells (72). Future studies of specific chain-length FOS, their catabolites, and their roles in healthy vs. dysbiotic intestinal microbiotas and healthy vs. inflamed intestinal states are warranted to better characterize the established prebiotic and putative pro-inflammatory properties of dietary fructans.

Although inulinase supplementation has the possibility of reducing GI symptoms by hydrolyzing fructans, the impacts of increased fructose availability should at least be considered. Fructose liberated from fructans in the stomach by inulinase are likely to be absorbed following transit to the upper intestine. In the intestine and other tissues, fructose absorption is regulated by expression of solute carrier family 2 member 5 (SLC2A5, also known as the glucose transporter GLUT5). The role of GLUT5, as the main fructose transporter, in health and disease has been broadly discussed (73–75). Excess fructose consumption that chronically increases blood fructose concentration has been associated with obesity, metabolic syndrome, type 2 diabetes, hypertension, and lipemia (75–77). However, these effects of fructose are only possible if plasma fructose can be absorbed in physiologically remarkable concentrations. The average intake of fructose in the USA has markedly increased in the past few decades, predominantly from the consumption of high-fructose corn syrups. Fructose consumption rates have steadily increased from an estimated 37 g/day in 1978 (78), to 49 g/day in 2004 (79), with the individuals in the 90% percentile consuming 75 g/day. Comparatively, mean intake of dietary fiber by adults in the US has remained low at 18.6 g/day (80). Moreover, inulin and FOS are typically found in foods below 5 g/serving (81). Altogether, increased fructose availability from inulinase supplementation is likely not to be substantial enough to approximate dietary fructose exposure associated with chronic health conditions. Nonetheless, we have included several blood markers of fructose exposure in a forthcoming human safety and tolerability trial of microbial inulinase supplmentation (51).

Our study comes with several limitations. While the static INFOGEST consensus method has been thoroughly validated (45, 46), and adapted to study supplemental microbial enzymes (42), it lacks the ability to alter pH in real time to approximate gastric pH when buffered by food, or as nutrients are released and become available for absorption. Acid-mediated hydrolysis of inulin did not occur under standard INFOGEST conditions, requiring protocol modifications to characterize the effects of increased gastric acidity (Figure 5). LC-MS profiling is expected to be accurate for inulins, however due to the limitations of hexose differentiation, fructan confirmation may be less accurate for complex substrates that include non-fructan sources of oligosaccharides, which may be the case in garlic and the FTM. These substrates are likely to contain short-chain GOS which have the same molar mass and may co-elute with fructans. Additionally, our experiments were limited to specific subsets of fructan-rich foods that did not include wheat and gluten. Note that the gluten-free peas and potatoes contribute to the carbohydrate content of the CTM, and there is no wheat “bun” for the black bean patty in the FTM. The intent was to specifically study fructans and develop substrates for clinical trials without confounding effects of gluten. While wheat and other cereals do contain fructans, it is now appreciated that gluten and fructans differentially modulate food intolerance. As such, adverse reactions typically associated with gluten consumption have been shown to be correlated with FODMAP consumption (82). For example, in a crossover study of IBS patients, a FODMAP challenge primarily consisting of fructose increased IBS symptom scores, however, a gluten challenge showed little difference from placebo (83). In individuals with non-celiac gluten sensitivity, fructans, rather than gluten, appear to be primary cause of GI symptoms (17). It has been suggested that a low FODMAP diet (i.e., fructan reduction) be the first approach to addressing wheat/gluten-related symptoms (84). Although newer processing methods can reduce the fructan content of wheat using inulinase (20, 85), no study has aimed to understand sensitivity to gluten after inulinase-mediated fructan hydrolysis. Future *in vitro* characterization of inulinase will include wheat-based substrates with and without other carbohydrases.

## 5. Conclusion

Results from this preclinical study of simulated fructan digestion support the efficacy of microbial inulinase in helping break down dietary fiber and reduce dietary FODMAP exposure. Inulinase treatment yielded significant hydrolysis of fructan-rich ingredients such as inulin and garlic at usual human gastric acidity *in vitro*. Differential effects of pH on acid-mediated fructan hydrolysis, independent of inulinase treatment, underscore the need to better understand the effects of different gastric acidities on fructan, fiber, and FODMAP digestion in vivo. Inulinase dose-ranging was broad enough to effectively inform dosing for a safety and tolerability trial in humans, as well as efficacy trials at lower doses.

## Data availability statement

The raw data supporting the conclusions of this article will be made available by the authors, without undue reservation.

## Author contributions

JG: conceptualization, methodology, formal analysis, visualization, and writing—original draft. MH: investigation, writing—reviewing and editing. JF: methodology, visualization, and writing—reviewing and editing. KT: conceptualization, methodology, supervision (supporting), and writing—reviewing and editing. SG: conceptualization (lead), supervision (lead), and writing—reviewing and editing. All authors contributed to the article and approved the submitted version.
