# Supplementary material for: Microbial inulinase promotes fructan hydrolysis under simulated gastric conditions
Source: Front Nutr. 2023 May 23;10:1129329. doi: 10.3389/fnut.2023.1129329 (PMC10251236; doi:10.3389/fnut.2023.1129329)
Supplement: Supplementary file 1 [file Data_Sheet_1.pdf]

SUPPLEMENTARY MATERIAL FOR:

Microbial inulinase promotes fructan hydrolysis under simulation gastric digestion

Justin L. Guice*, Morgan D. Hollins, James G. Farmar, Kelly M. Tinker, Sean M. Garvey*

Department of Research and Development, BIO-CAT, Inc.,
9117 Three Notch Rd, Troy, VA 22974, USA

*Corresponding authors at: Department of Research and Development, BIO-CAT, Inc.,
9117 Three Notch Rd, Troy, VA 22974, USA.

E-mail addresses: sgarvey@bio-cat.com (S.M. Garvey), jguice@bio-cat.com (J.L. Guice)

© 2023 BIO-CAT, Inc.

Table S1. Proximate analysis of the high FODMAP test meal

FTM composition			
Prepared item	Amount	Units	Ingredients
Brussels sprout	45.00	g	Brussels sprout, cooked
Black Bean Patty	100.00	g	
Garlic	5.00	g	Garlic, raw
Onion	46.00	g	Onion, white, raw
Olive Oil	5.00	g	Oil, olive, extra virgin
Black bean patty composition			
Black beans	260.00	g	Black beans, canned, reduced sodium
Bell pepper	60.00	g	Peppers, sweet, green, raw
Bread crumbs	55.00	g	Plain bread crumbs, plain, gluten free
Flaxseed meal	8.00	g	Golden flaxseed meal
Onion	115.00	g	Onion, white, raw
Garlic powder	15.00	g	Spices, garlic powder
Onion powder	25.00	g	Spices, onion powder
Chili powder	6.00	g	Spices, chili powder
Cumin	2.50	g	Spices, cumin seed
Salt	1.50	g	Salt, table, iodized
Water	40.00	mL	Beverages, water, tap, drinking
Nutrition profile by weight*			
Analyte	Results	Units	Method
Calories	142.00	kcal/100g	Calculated
Moisture	67.43	g/100g	AOAC 925.10 (mod.)
Protein	5.05	g/100g	AOAC 2001.11 (mod.)
Sodium	171.33	mg/100g	AOAC 2011.14 (mod.)
Total carbohydrates	22.57	g/100g	Calculated
Total dietary fiber†	5.80	g/100g	AOAC 991.43 (mod.)
Total fat	3.44	g/100g	AOAC 922.06 (mod.)
Nutrition profile by meal*			
Analyte	Results	Units	
Calories	285.42	kcal	
Moisture	135.34	g	
Protein	10.15	g	
Sodium	344.38	mg	
Total carbohydrates	45.36	g	
Total dietary fiber†	11.66	g	
Total Fat	6.91	g	

Abbreviations:

FTM, high FODMAP test meal; AOAC, Association of Official Agricultural Chemists

Footnotes:

*Proximate analytes are an average of three separately prepared meals

†Total dietary fiber is an average of two randomly selected meals

© 2023 BIO-CAT, Inc.

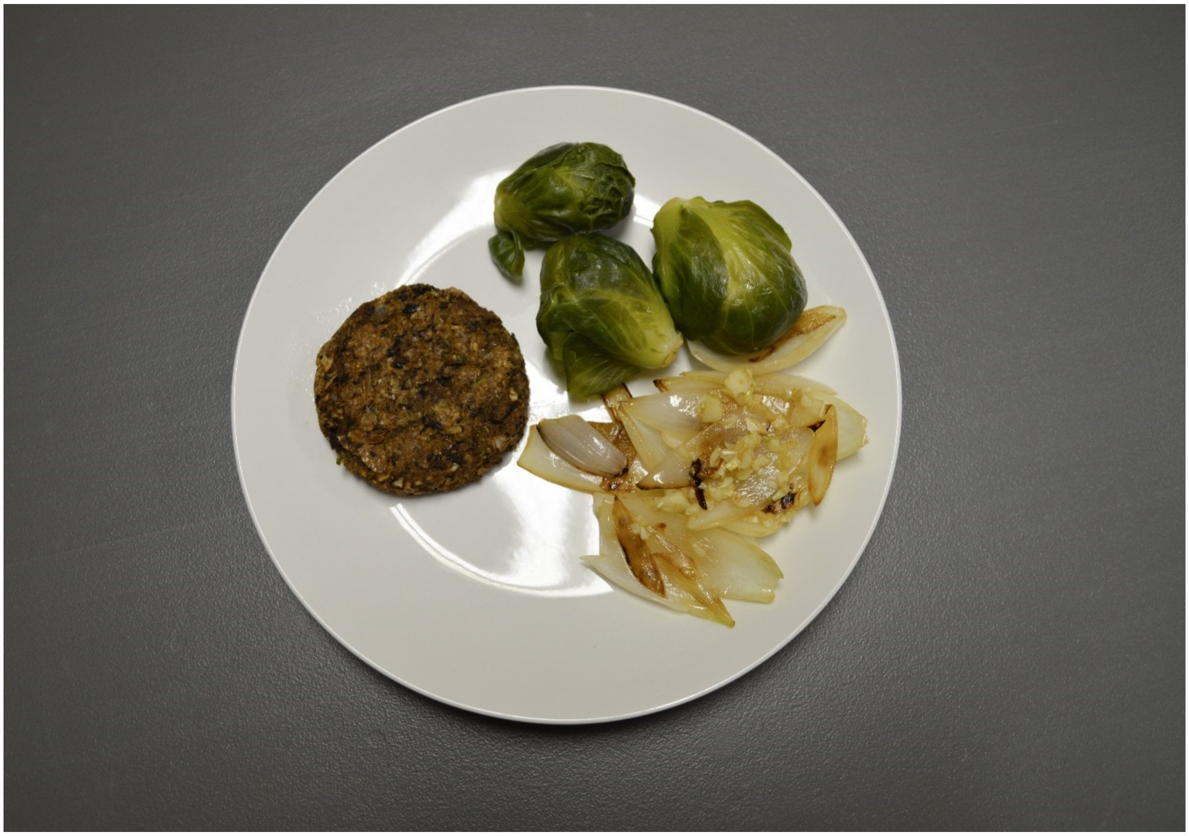

Fig. S1. High FODMAP test meal (FTM) comprised of a cooked black bean patty, steamed Brussels sprouts, and sautéed garlic and onions.

© 2023 BIO-CAT, Inc.

Table S2. Inulin-derived hexose sugar mass database created with Agilent MassHunter PDCL Manager

DP	Formula	Mass (m/z)
DP1	$C_6H_{12}O_6$	180.0634
DP2	$C_{12}H_{22}O_{11}$	342.1162
DP3	$C_{18}H_{32}O_{16}$	504.1690
DP4	$C_{24}H_{42}O_{21}$	666.2219
DP5	$C_{30}H_{52}O_{26}$	828.2747
DP6	$C_{36}H_{62}O_{31}$	990.3275
DP7	$C_{42}H_{72}O_{36}$	1152.3803
DP8	$C_{48}H_{82}O_{41}$	1314.4332
DP9	$C_{54}H_{92}O_{46}$	1476.4860
DP10	$C_{60}H_{102}O_{51}$	1638.5388
DP11	$C_{66}H_{112}O_{56}$	1800.5916
DP12	$C_{72}H_{122}O_{61}$	1962.6445
DP13	$C_{78}H_{132}O_{66}$	2124.6973
DP14	$C_{84}H_{142}O_{71}$	2286.7501
DP15	$C_{90}H_{152}O_{76}$	2448.8029
DP16	$C_{96}H_{162}O_{81}$	2610.8557
DP17	$C_{102}H_{172}O_{86}$	2772.9086
DP18	$C_{108}H_{182}O_{91}$	2934.9614
DP19	$C_{114}H_{192}O_{96}$	3097.0142
DP20	$C_{120}H_{202}O_{101}$	3259.0670
DP21	$C_{126}H_{212}O_{106}$	3421.1199
DP22	$C_{132}H_{222}O_{111}$	3583.1727
DP23	$C_{138}H_{232}O_{116}$	3745.2255
DP24	$C_{144}H_{242}O_{121}$	3907.2783
DP25	$C_{150}H_{252}O_{126}$	4069.3312
DP26	$C_{156}H_{262}O_{131}$	4231.3840
DP27	$C_{162}H_{272}O_{136}$	4393.4368
DP28	$C_{168}H_{282}O_{141}$	4555.4896
DP29	$C_{174}H_{292}O_{146}$	4717.5424
DP30	$C_{180}H_{302}O_{151}$	4879.5953
DP31	$C_{186}H_{312}O_{156}$	5041.6481
DP32	$C_{192}H_{322}O_{161}$	5203.7009
DP33	$C_{198}H_{332}O_{166}$	5365.7537
DP34	$C_{204}H_{342}O_{171}$	5527.8066
DP35	$C_{210}H_{352}O_{176}$	5689.8594
DP36	$C_{216}H_{362}O_{181}$	5851.9122
DP37	$C_{222}H_{372}O_{186}$	6013.9650
DP38	$C_{228}H_{382}O_{191}$	6176.0179
DP39	$C_{234}H_{392}O_{196}$	6338.0707
DP40	$C_{240}H_{402}O_{201}$	6500.1235
DP41	$C_{246}H_{412}O_{206}$	6662.1763
DP42	$C_{252}H_{422}O_{211}$	6824.2292
DP43	$C_{258}H_{432}O_{216}$	6986.2820
DP44	$C_{264}H_{442}O_{221}$	7148.3348
DP45	$C_{270}H_{452}O_{226}$	7310.3876
DP46	$C_{276}H_{462}O_{231}$	7472.4404
DP47	$C_{282}H_{472}O_{236}$	7634.4933
DP48	$C_{288}H_{482}O_{241}$	7796.5461
DP49	$C_{294}H_{492}O_{246}$	7958.5989
DP50	$C_{300}H_{502}O_{251}$	8120.6517

Abbreviations: DP, degree of polymerization

© 2023 BIO-CAT, Inc.

Table S3. Fructose concentrations of gastric digestas following simulated salivary-gastric (SG) digestion of inulin, garlic, inulin-spiked CTM, garlic-spiked CTM, and FTM[†]

	Experimental conditions Fructose (mg/g)										ANOVA (F) p-value
**Inulin**											
	Control	3.13 INU	6.25 INU	12.5 INU	25 INU	50 INU	100 INU	200 INU	400 INU	800 INU	
Mean	34.8[a]	88.9[b]	85.6[b]	140.6[c]	197.0[d]	307.1[e]	347.7[f]	412.7[g]	434.6[g]	421.2[g]	< 0.0001
± SD	8.18	12.19	5.75	9.00	5.08	4.71	24.45	8.48	12.36	4.57	
Fold-change	-	2.6	2.5	4.0	5.7	8.8	10.0	11.9	12.5	12.1	
**Garlic**											
	Control	3.13 INU	6.25 INU	12.5 INU	25 INU	50 INU	100 INU	200 INU	400 INU	800 INU	
Mean	20.7[a]	46.6[b]	59.2[c]	76.6[d]	110.6[e]	142.1[f]	161.0[g]	174.7[h]	180.0[hi]	189.7[i]	< 0.0001
± SD	1.93	1.64	3.63	3.98	3.35	9.47	2.71	4.54	1.60	3.45	
Fold-change	-	2.3	2.9	3.7	5.4	6.9	7.8	8.5	8.7	9.2	
**iCTM (CTM + inulin)**											
	Control					50 INU	100 INU	200 INU	400 INU	800 INU	
Mean	2.82[a]	-	-	-	-	38.24[b]	51.38[c]	57.58[d]	59.28[d]	63.14[e]	< 0.0001
± SD	0.29	-	-	-	-	0.60	1.15	1.27	2.07	1.97	
Fold-change	-	-	-	-	-	13.6	18.2	20.4	21.0	22.4	
**gCTM (CTM + garlic)**											
	Control					50 INU	100 INU	200 INU	400 INU	800 INU	
Mean	8.173[a]	-	-	-	-	58.49[b]	73.43[c]	79.56[cd]	81.99[cd]	83.78[d]	< 0.0001
± SD	0.43	-	-	-	-	4.83	2.90	3.50	6.11	1.33	
Fold-change	-	-	-	-	-	7.2	9.0	9.7	10.0	10.3	
**FTM**											
	Control					50 INU	100 INU	200 INU	400 INU	800 INU	
Mean	10.11[a]	-	-	-	-	25.33[b]	27.77[c]	29.96[d]	31.93[de]	33.27[e]	< 0.0001
± SD	0.27	-	-	-	-	0.11	0.95	0.20	1.44	0.61	
Fold-change	-	-	-	-	-	2.5	2.7	3.0	3.2	3.3	

Abbreviations: CTM, canned test meal; FTM, high FODMAP test meal; gCTM, garlic-spiked test meal; iCTM, inulin-spiked canned test meal; SD, standard deviation; SG, salivary-gastric

Footnotes:

[†]Each measurement is the average of three independent replicates and is expressed as means ± SD. Significant differences between samples are denoted by unshared lower-case letters (a, b, c, d, e, f, g, h, i).

© 2023 BIO-CAT, Inc.

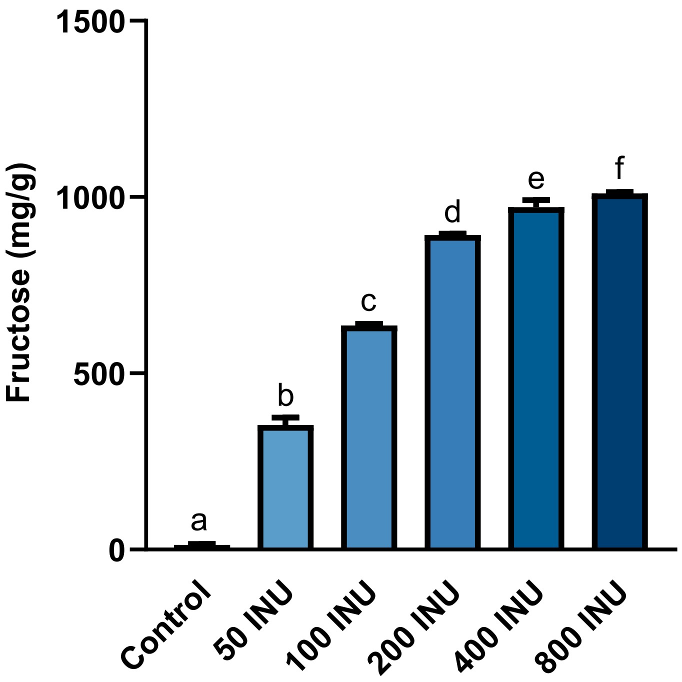

Fig. S2. Fructose concentrations of gastric digestas after inulinase treatment of inulin from chicory (Sigma-Aldrich) under standard INFOGEST salivary-gastric conditions ($n = 3$). Error bars show ± 1 standard deviation. Significant differences ($p < 0.05$) between samples are denoted by unshared lower-case letters (a, b, c, d, e). INU, inulinase activity unit.

© 2023 BIO-CAT, Inc.

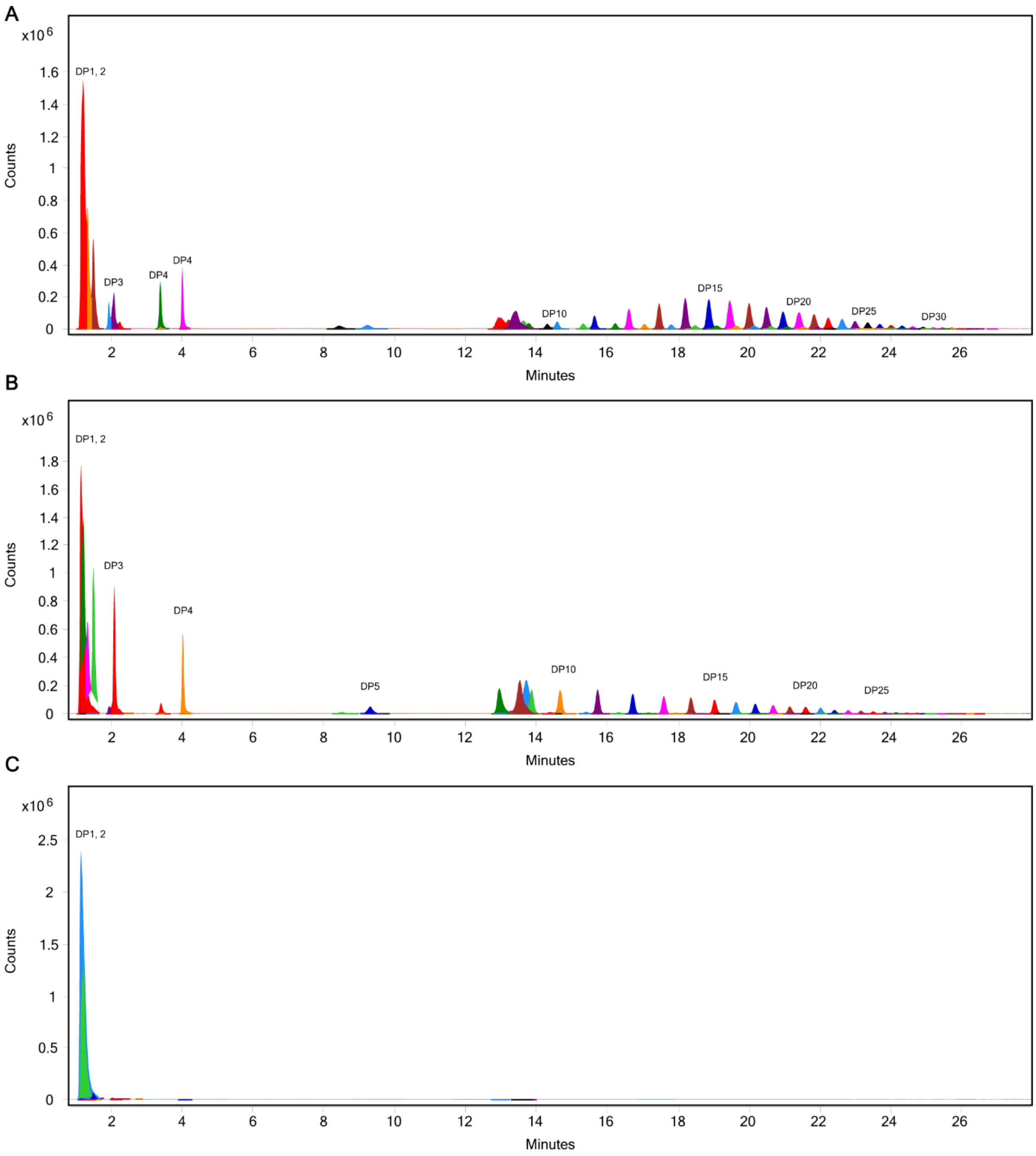

Fig. S3. Overlaid extracted ion chromatograph of inulooligosaccharides (IOS) following simulated salivary-gastric digestion of "inulin from chicory" under standard INFOGEST conditions, (**A**) Control, (**B**) 50 INU/serving, (**C**) 400 INU/serving.

© 2023 BIO-CAT, Inc.

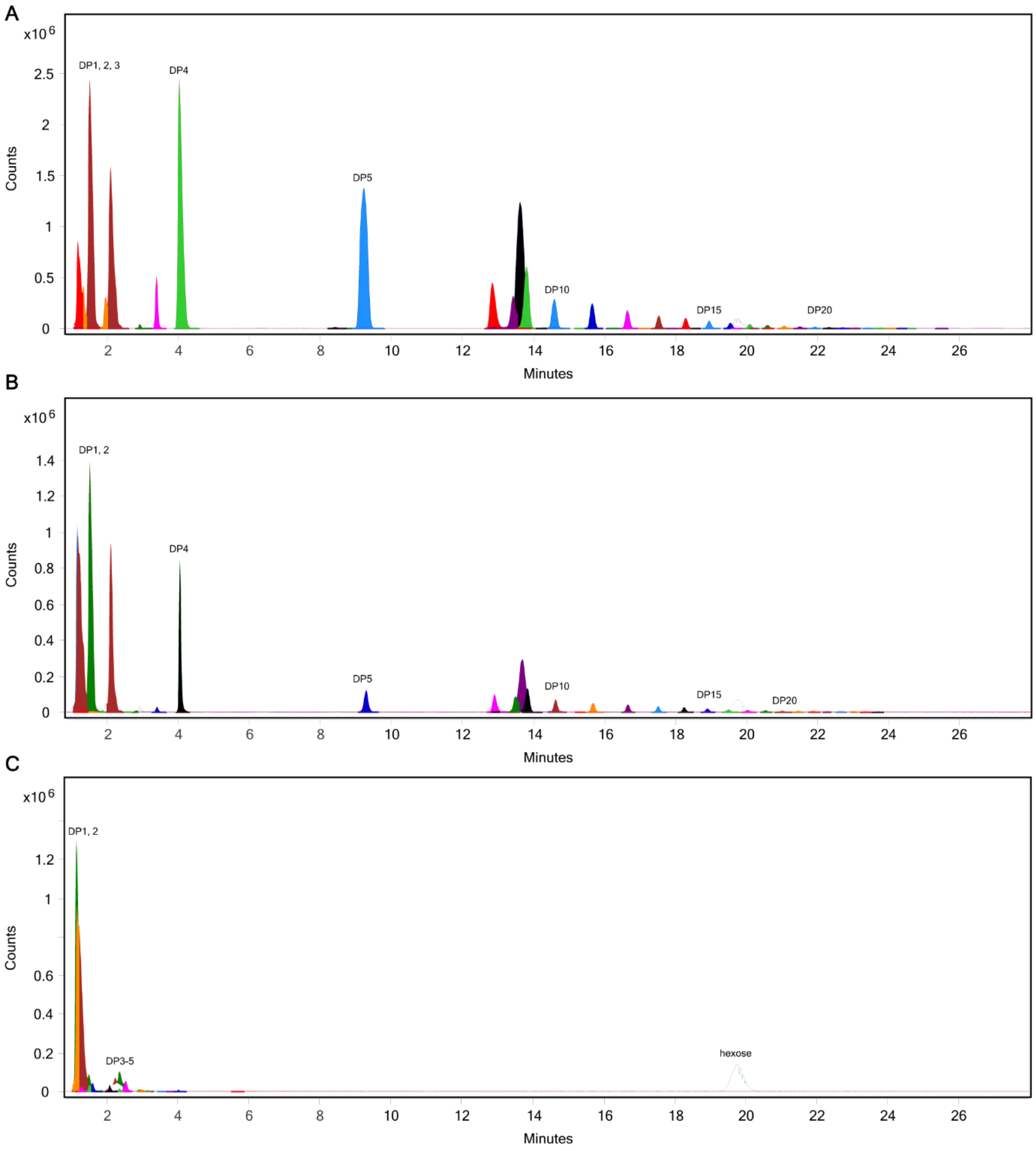

Fig. S4. Overlaid extracted ion chromatograph of fructooligosaccharides (FOS) following simulated salivary-gastric digestion of a standardized canned test meal (CTM) spiked with inulin (iCTM) under standard INFOGEST conditions, (**A**) Control, (**B**) 50 INU/serving, (**C**) 400 INU/serving.

© 2023 BIO-CAT, Inc.

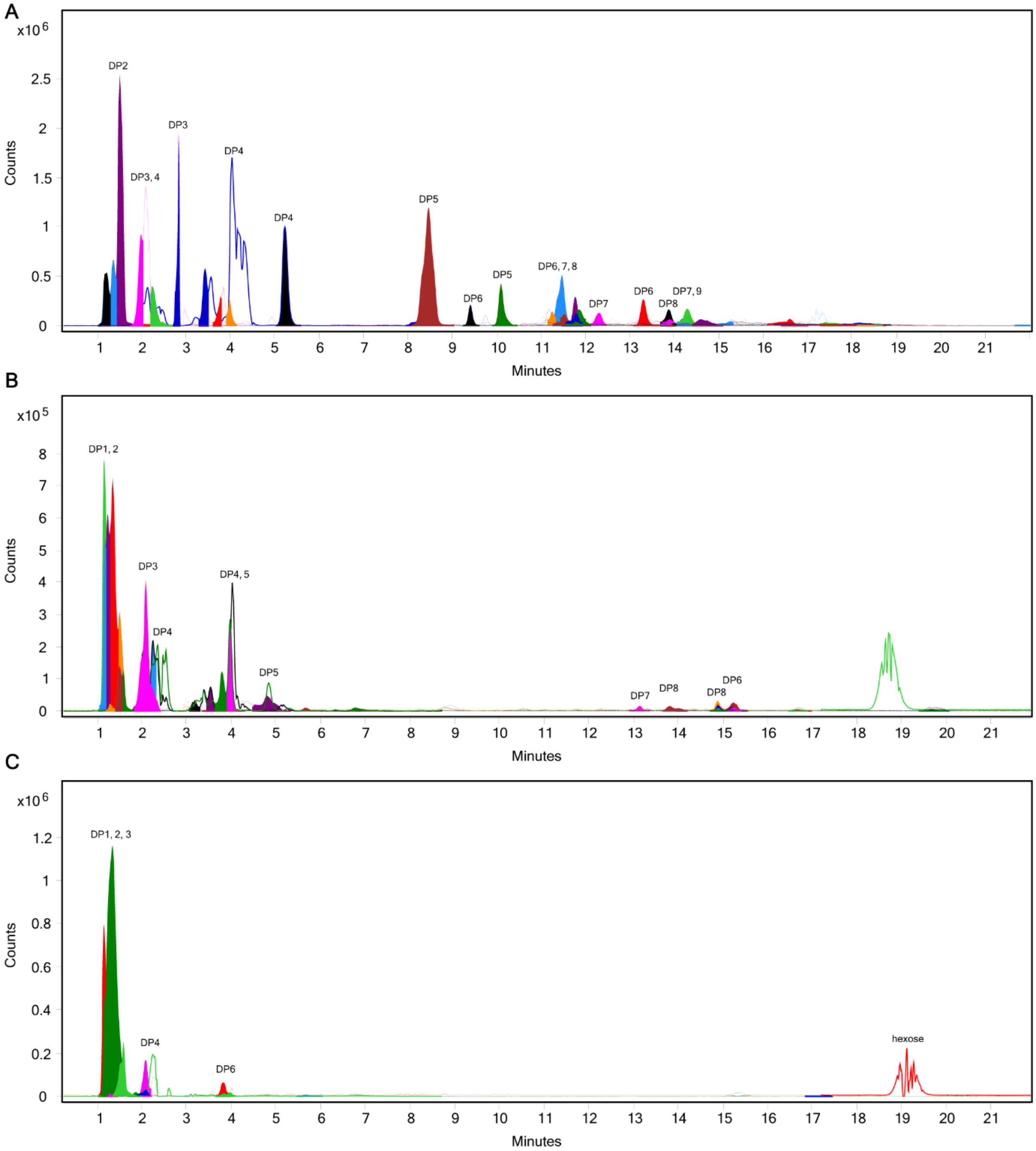

Fig. S5. Overlaid extracted ion chromatograph of fructooligosaccharides (FOS) following simulated salivary-gastric digestion of a standardized canned test meal (CTM) spiked with garlic (gCTM) under standard INFOGEST conditions, (**A**) Control, (**B**) 50 INU/serving, (**C**) 400 INU/serving.

© 2023 BIO-CAT, Inc.
